# The Intersection of Glycosylation and Ferroptosis in Cancer

**DOI:** 10.3390/antiox14091077

**Published:** 2025-09-02

**Authors:** Jihan Kim, Junghyun Kim, Man S. Kim

**Affiliations:** 1 Translational-Transdisciplinary Research Center, Medical Science Research Institute, Kyung Hee University Hospital at Gangdong, College of Medicine, Kyung Hee University, Seoul 05278, Republic of Korea; kknowing@khu.ac.kr; 2 Department of Medicine, Kyung Hee University College of Medicine, Seoul 02453, Republic of Korea; 3 Division of Tourism & Wellness, Hankuk University of Foreign Studies, Yongin-si 17035, Gyeonggi-do, Republic of Korea; jh.kim@hufs.ac.kr

**Keywords:** ferroptosis, glycosylation, cancer, oxidative stress

## Abstract

Ferroptosis, an iron-dependent form of regulated cell death characterized by lipid peroxidation, has emerged as a critical mechanism in cancer biology and therapy. Aberrant glycosylation is a hallmark of cancer, influencing cellular processes from proliferation to immune evasion. Recent evidence has revealed previously underappreciated crosstalk between glycosylation and ferroptosis in cancer cells, where specific glycosylation modifications can determine cellular susceptibility to ferroptotic cell death. This review summarizes the current understanding of how N-linked glycosylation, O-linked glycosylation, and glycosaminoglycan biosynthesis modulate sensitivity to ferroptosis in various cancers. We examine the molecular mechanisms underlying glycosylation-dependent ferroptosis regulation, including the roles of key glycosyltransferases and glycan structures in the oxidative stress response. Furthermore, we discuss the therapeutic potential of targeting the glycosylation–ferroptosis axis for cancer treatment in this emerging field.

## 1. Introduction

Cancer cells exhibit remarkable metabolic plasticity that enables their survival under diverse stress conditions [1]. Among the various forms of regulated cell death, ferroptosis has garnered significant attention as a potential therapeutic target owing to its distinct characteristics and relevance in cancer biology [2]. Ferroptosis is an iron-dependent form of regulated cell death driven by the accumulation of lipid peroxides and subsequent membrane damage. Unlike apoptosis, necroptosis, and autophagy, ferroptosis is characterized by unique morphological features, including mitochondrial shrinkage, increased membrane density, and the absence of chromatin condensation [3]. Central to ferroptosis execution is the role of oxidative stress, defined as an imbalance between reactive oxygen species (ROS) generation and antioxidant defenses [4]. Cancer cells frequently exhibit elevated basal ROS levels due to oncogenic signaling and metabolic reprogramming, placing them in a precarious redox state that predisposes them to lipid peroxidation and ferroptotic cell death [5]. This oxidative vulnerability represents both a cancer cell weakness and a therapeutic opportunity, as the disruption of antioxidant systems can tip the balance toward ferroptotic demise.

The regulation of ferroptosis involves complex molecular networks centered around iron metabolism, lipid peroxidation, and antioxidant defense systems [6]. Glutathione peroxidase 4 (GPX4) serves as the central guardian of ferroptosis by reducing lipid peroxides to their corresponding alcohols [7]. The cystine/glutamate antiporter system Xc^−^ is composed of the heavy chain subunit 4F2hc, encoded by SLC3A2, and light chain subunit xCT, encoded by SLC7A11. It maintains cellular glutathione levels essential for GPX4 function [8]. Additional ferroptosis suppressors include ferroptosis suppressor protein 1 (FSP1) and the CoQ10/vitamin K pathway [9,10,11].

Recent studies have identified several additional ferroptosis regulators, including dihydroorotate dehydrogenase, which acts as a mitochondrial suppressor through its effects on mitochondrial CoQ10 levels [12], and 7-dehydrocholesterol, which acts as an endogenous ferroptosis suppressor [13,14]. The discovery of sex-dependent ferroptosis defense pathways has added another layer of complexity to our understanding of the regulation of ferroptosis [15].

Glycosylation is one of the most complex and diverse post-translational modifications and affects protein folding, stability, localization, and function [16]. In cancer, aberrant glycosylation contributes to virtually all the hallmarks of the disease, including sustained proliferation, immune evasion, angiogenesis, and metastasis [17]. The two major forms of protein glycosylation, N-linked glycosylation at asparagine residues and O-linked glycosylation at serine/threonine residues, are catalyzed by specific glycosyltransferases in the endoplasmic reticulum and Golgi apparatus [18]. Glycosaminoglycans (GAGs) are a specialized class of glycan structures that play a crucial role in cell surface interactions and signaling [19].

The clinical relevance of glycosylation in cancer is well established, with aberrant glycosylation patterns serving as biomarkers for diagnosis and prognosis of cancer [20,21]. Unsurprisingly, the development of glycan-based therapeutics, including glycosylation inhibitors and immunotherapies targeting tumor-associated carbohydrate antigens, has gained significant momentum [22,23,24].

Despite extensive research on ferroptosis and glycosylation in cancer, their intersection has only recently emerged as a significant area of study. Although evidence suggests that glycosylation influences cellular responses to oxidative stress [25,26], the molecular crosstalk between altered glycosylation and ferroptotic sensitivity in cancer remains poorly understood.

**Figure 1:**
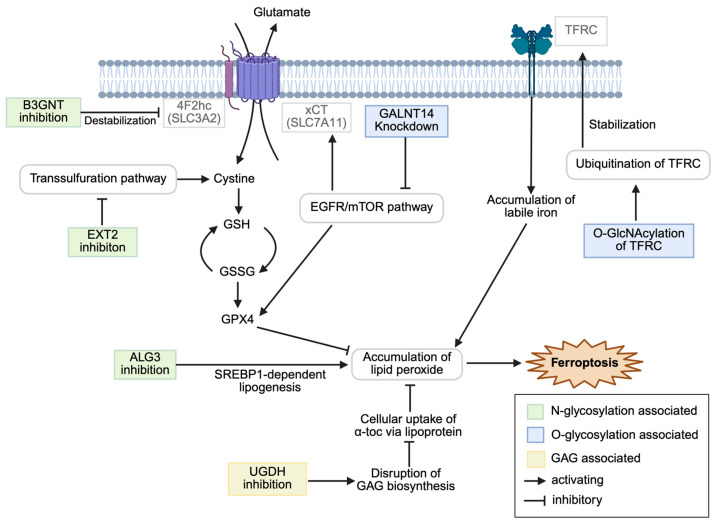
Schematic overview of glycosylation-associated modulation of ferroptosis in cancer cells. α-toc, α-tocopherol; EGFR, epidermal growth factor receptor; GAG, glycosaminoglycan; GlcNAcylation, N-acetylglucosaminylation; SREBP, sterol-regulated element-binding protein; TFRC, transferrin receptor. Created in BioRender. CMOL. (2025) https://BioRender.com/tlo17mi (accessed on 25 July 2025).

This review aims to summarize the current knowledge on the intersection of glycosylation and ferroptosis in cancer, examine the molecular mechanisms by which specific glycosylation modifications modulate ferroptosis sensitivity, and explore the therapeutic implications of targeting this axis for cancer treatment. Figure 1 summarizes the modulation of ferroptosis.

## 2. Molecular Mechanisms of Ferroptosis and Glycosylation

### 2.1. Oxidative Stress and ROS in Ferroptosis Regulation

Ferroptosis is a regulated form of cell death distinguished by iron-dependent accumulation of lipid peroxides, setting it apart from apoptosis, necroptosis, and other cell-death modalities [2]. Oxidative stress—defined as an imbalance between ROS generation and antioxidant defenses—plays a pivotal role in triggering ferroptosis by overwhelming cellular redox systems [4,27]. Cancer cells frequently exhibit elevated basal ROS due to oncogenic signaling and metabolic reprogramming, placing them on a narrow redox fulcrum that predisposes them to lipid peroxidation and ferroptotic demise [5].

Mechanistically, ferroptosis is driven by iron-catalyzed peroxidation of polyunsaturated fatty acids (PUFAs) in membrane phospholipids. Glutathione peroxidase 4 (GPX4) serves as a central safeguard by reducing lipid hydroperoxides to non-toxic alcohols; its inhibition or depletion leads to unchecked peroxidation and cell death [7]. The acyl-CoA synthetase long-chain family member 4 (ACSL4) further shapes ferroptosis sensitivity by enriching membranes with PUFA-containing phospholipids that are prime substrates for ROS-mediated attack [28]. Oxidative stress exacerbates ferroptosis by depleting NADPH reserves required for glutathione regeneration via the pentose phosphate pathway, coupling central carbon metabolism with ferroptotic execution [29].

Sources of cellular ROS include mitochondrial electron transport-chain leakage, NADPH oxidase complexes, and peroxisomal metabolism. Under normal physiology, antioxidant networks—comprising catalase, superoxide dismutases, and the glutathione–GPX4 axis—maintain redox homeostasis [4,5]. In cancer, metabolic reprogramming driven by oncogenes elevates mitochondrial ROS generation, which can paradoxically fuel proliferation yet prime cells for oxidative collapse [4]. The p53 tumor suppressor exploits this vulnerability by transcriptionally repressing the cystine/glutamate antiporter xCT, thereby diminishing glutathione levels and amplifying ROS-induced lipid peroxidation to induce ferroptosis [3]. Additionally, interferon-γ is released by clusters of differentiation 8 (CD8)^+^ T cells and downregulates xCT within the tumor microenvironment, impairing cystine import and heightening oxidative stress that culminates in ferroptotic cell death [30].

### 2.2. Protein Glycosylation: Fundamentals and Cellular Functions

Protein glycosylation represents one of the most complex and ubiquitous post-translational modifications in eukaryotic cells, affecting an estimated 50% or more of all mammalian proteins [31,32]. This sophisticated process involves the covalent attachment of carbohydrate groups (glycans) to specific amino acid residues on proteins, fundamentally altering their structure, function, and biological properties [33,34]. The importance of glycosylation extends far beyond simple structural decoration, as it serves as a critical regulatory mechanism governing protein folding, stability, localization, trafficking, and intermolecular interactions [33,35].

The glycosylation machinery represents a remarkable example of biological complexity, requiring the coordinated action of over 200 different enzymes, including glycosyltransferases, glycosidases, and associated regulatory proteins [36]. These enzymes work in concert within the endoplasmic reticulum (ER) and Golgi apparatus to synthesize, modify, and process glycan structures with extraordinary precision and diversity [37]. The process begins in the ER, where the initial steps of N-linked glycosylation occur through the action of oligosaccharyltransferase complexes, and continues through the Golgi apparatus, where extensive glycan remodeling takes place through the sequential action of various glycosyltransferases and glycosidases [38].

The biological importance of protein glycosylation extends across virtually all aspects of cellular function and organism development. For example, protein folding and quality control represent fundamental roles of glycosylation, particularly in the ER, where N-linked glycans serve as recognition elements for molecular chaperones and folding sensors [39]. The calnexin/calreticulin system recognizes monoglucosylated N-glycans on nascent glycoproteins, facilitating proper folding and preventing the export of misfolded proteins from the ER [40].

This complex and highly regulated system of protein glycosylation provides the foundation for understanding how specific glycosyltransferases and glycan structures contribute to cellular processes such as ferroptosis regulation. In this review, genes and/or proteins associated with the regulation of ferroptosis through glycosylation will be discussed, and they are organized in Table 1.

## 3. N-Linked Glycosylation and Ferroptosis Regulation

### 3.1. 4F2hc-B3GNT3 Axis in Pancreatic Cancer

Recent glycoproteomic investigations have identified β-1,3-N-acetylglucosaminyltransferase 3, encoded by B3GNT3, as a critical regulator of ferroptosis resistance in pancreatic ductal adenocarcinoma (PDAC) through its N-glycosylation of 4F2hc, the heavy chain subunit of the glutamate/cystine antiporter system Xc^−^ [41]. B3GNT3-mediated glycosylation stabilizes 4F2hc and enhances its interaction with xCT to form the functional system Xc^−^ complex, which is essential in cystine uptake and glutathione biosynthesis [8,52]. Knockdown of B3GNT3 significantly sensitizes PDAC cells to ferroptosis by impairing system Xc^−^ activity, leading to decreased intracellular glutathione levels and accumulation of lipid peroxides [2,41].

Compared with its non-glycosylated form, glycosylated 4F2hc shows enhanced membrane localization, which in turn facilitates the stability and functional expression of the associated xCT subunit [41]. The 4F2hc-xCT interaction occurs at different cellular regions through various interactions: polar interactions in the extracellular space and hydrophobic interactions within the transmembrane region [53]. Additionally, the short helix formed by the transmembrane helix of 4F2hc contributes to system Xc^−^ structural stability by anchoring xCT to the intracellular side [53]. Reconstitution experiments demonstrated that enzymatically active B3GNT3 rescued ferroptosis sensitivity in the knockout cells, confirming the direct role of glycosyltransferase activity in ferroptosis resistance [41]. This mechanism is particularly critical in PDAC cells that experience elevated oxidative stress due to metabolic reprogramming and oncogenic signaling [54].

The therapeutic implications are significant, as high expression levels of both 4F2hc and B3GNT3 correlate with disease progression and poor survival in PDAC patients [55]. Combination treatment with classical N-glycosylation inhibitors such as tunicamycin enhanced ferroptosis sensitivity in PDAC cells, suggesting that targeting glycosylation is a promising therapeutic strategy when combined with ferroptosis inducers [41]. The B3GNT3-4F2hc axis thus represents a novel therapeutic vulnerability to this aggressive malignancy that notoriously resists conventional treatments.

### 3.2. ALG3-Mediated N-Glycosylation Deficiency and Immunogenic Ferroptosis

Alpha-1,3-mannosyltransferase 3, encoded by ALG3, represents another critical node in the N-glycosylation–ferroptosis axis [42]. ALG3 catalyzes the first dolichol–phosphate–mannose-dependent mannosylation step in the biosynthesis of lipid-linked oligosaccharides, which are essential precursors for protein N-glycosylation [56,57]. The early position of the enzyme in the N-glycan assembly pathway makes it particularly important for overall glycosylation capacity [38].

The mechanism underlying ALG3-dependent ferroptosis regulation involves complex dysregulation of lipid metabolism. N-glycosylation deficiency resulting from ALG3 inhibition leads to excessive lipid accumulation via sterol-regulated element-binding protein 1 (SREBP1)-dependent lipogenesis in renal cortical adenocarcinoma (Renca), colorectal adenocarcinoma (MC38), melanoma (B16), and breast cancer (4T1) [42]. This disruption of metabolic homeostasis creates a cellular environment prone to lipid peroxidation because cancer cells are unable to maintain a proper lipid balance [7, 58]. The resulting lipid hyperaccumulation provides abundant substrates for ferroptotic cell death via enhanced peroxidation [1].

Importantly, ferroptosis induced by ALG3 inhibition exhibits distinct immunogenic characteristics, including the release of damage-associated molecular patterns that promote anti-tumor immune responses [6,42]. Immunogenic cell death is characterized by the exposure to calreticulin on the cell surface, release of high-mobility group box 1, and secretion of ATP, all of which serve as signals to activate dendritic cells and promote T-cell responses [59,60]. The connection between glycosylation defects and immunogenic cell death represents a novel mechanism by which metabolic stress enhances tumor immunogenicity [61].

The therapeutic implications of ALG3 targeting are particularly compelling because ALG3 inhibition synergizes with immune checkpoint blockade therapy in mouse cancer models [42,62]. Treatment with tunicamycin, a broad N-glycosylation inhibitor that affects multiple steps in the pathway, including ALG3-dependent processes, produces effects similar to those of ALG3 deletion, suggesting that general N-glycosylation deficiency can promote ferroptosis-mediated tumor suppression [42,63]. These findings highlight the potential of combining more selective glycosylation inhibitors with immunotherapeutic approaches.

### 3.3. EXT2-Mediated Transsulfuration and Diminished Antioxidant Capacity

Exostosin glycosyltransferase 2, encoded by EXT2, forms a heterodimeric complex with EXT1 in the Golgi apparatus, which catalyzes heparan sulfate chain polymerization through the alternating transfer of N-acetylglucosamine and glucuronic acid residues [64,65]. Recent structural studies have revealed that EXT2 primarily contributes to N-acetylglucosamine transferase activity, whereas EXT1 provides both glycosyltransferase activities required for heparan sulfate backbone synthesis [65,66]. Nadanaka et al. reported the roles of exostosin-like glycosyltransferase 2 (EXTL2), a member EXT family, implying that the metabolic effects of EXT2 depletion may be linked to disruption of GAG biosynthesis, which requires substantial metabolic resources and competes with other cellular pathways [67].

Recent investigations have identified EXT2 as a novel regulator of ferroptosis sensitivity in glioblastoma owing to its unexpected effects on cellular metabolism [43]. EXT2 depletion in glioblastoma cells results in reduced viability, enhanced radiosensitivity, and increased susceptibility to ferroptosis. Mechanistically, EXT2-depleted cells exhibit altered S-adenosylmethionine metabolism and decreased levels of transsulfuration pathway metabolites, leading to diminished antioxidant capacity and enhanced lipid peroxidation [43]. This pathway provides an alternative source of cysteine for glutathione synthesis, independent of the system Xc^−^ antiporter [68,69].

The identification of EXT2 as a regulator of ferroptosis has significant therapeutic implications for glioblastoma treatment. Many glioblastoma cells resist system Xc^−^ inhibitors such as erastin due to their ability to obtain cysteine through the transsulfuration pathway [70,71]. Targeting EXT2 and compromising transsulfuration pathway activity may help to overcome this resistance and sensitize cells to ferroptosis. Combined approaches involving EXT2 inhibition and dietary methionine and cysteine restriction could potentially achieve synergistic anti-tumor effects by limiting substrate availability for alternative cysteine synthesis pathways [40,59,71,72].

### 3.4. Additional N-Glycosylation Regulators

In addition to the well-characterized examples of B3GNT3, ALG3, and EXT2, several other N-glycosylation-related genes and pathways have been implicated in the regulation of ferroptosis through diverse molecular mechanisms. The endoplasmic reticulum stress response, which is intricately linked to protein glycosylation quality control, is a major regulatory node that modulates ferroptosis sensitivity through multiple interconnected pathways [40,72].

The unfolded protein response, which is activated by the accumulation of misfolded or unglycosylated proteins in the ER, influences ferroptosis by regulating antioxidant gene expression and cellular metabolism [39,40]. The three arms of the unfolded protein response—PERK, IRE1α, and ATF6—are implicated in ferroptosis regulation, either directly or indirectly, through their effects on the glycosylation machinery.

## 4. O-Linked Glycosylation in Ferroptosis Modulation

### 4.1. O-GlcNAcylation-Mediated Ferroptosis Sensitivity

O-linked β-N-acetylglucosaminylation (O-GlcNAcylation), catalyzed by O-GlcNAc transferase (OGT), represents a unique form of protein glycosylation that differs markedly from the classical N-linked and mucin-type O-linked glycosylation discussed previously [35,73]. This dynamic post-translational modification involves the addition of a single N-acetylglucosamine sugar moiety to serine and threonine residues of nuclear, cytoplasmic, and mitochondrial proteins, catalyzed by O-GlcNAc transferase and removed by O-GlcNAcase [74].

In acute liver injury, ferroptosis in hepatocytes is suppressed by the ubiquitination of transferrin receptor (TFRC), which promotes its degradation and limits iron accumulation. Wu and colleagues demonstrated that the HECT domain-containing ubiquitin ligase E3 ligase (HUWE1) specifically targets TFRC for proteasomal degradation, thereby protecting hepatocytes from ferroptotic cell death during acute liver injury [45]. In hepatocellular carcinoma (HCC), one of the most significant breakthroughs in exemplifying O-GlcNAcylation–ferroptosis interactions came from Zhou and colleagues’ discovery that TFRC undergoes O-GlcNAcylation, which plays a role in erastin-induced ferroptosis sensitivity [44]. When ferroptosis is induced by erastin, it triggers de-O-GlcNAcylation at serine 687 (Ser687) of TFRC, leading to diminished binding of the ubiquitin E3 ligase to membrane-associated RING-CH8 (MARCH8) and decreased polyubiquitination at lysine 665 (Lys665). It also results in the enhancement of TFRC stability, which promotes labile iron accumulation, thereby sensitizing cells to ferroptotic cell death [44]. This mechanism represents a novel paradigm, where O-GlcNAcylation functions as a molecular switch that can be dynamically regulated to control iron homeostasis and ferroptosis sensitivity. Moreover, O-GlcNAcylation of yes-associated protein (YAP) elevated the transcriptional level of TFRC and increased the sensitivity in HCC [46].

The pivotal role of O-GlcNAcylation for ferroptosis modulation in HCC can be significant. The dynamic nature of O-GlcNAcylation may make it an attractive target for therapeutic intervention. Modulation of OGT activity could potentially sensitize resistant HCC cells to ferroptosis-inducing therapies, while the identification of specific O-GlcNAcylation sites on ferroptosis regulators like TFRC provides opportunities for developing targeted small-molecule inhibitors that selectively disrupt these protective modifications.

### 4.2. Mucin-Type O-Glycosylation and Cell Surface Modifications

In addition to specific glycosyltransferases, the broader landscape of mucin-type O-glycosylation influences cell surface properties and receptor function [75].

Recent studies have revealed a direct mechanistic link between mucin-type O-glycosylation and ferroptosis in cancer cells. The most compelling evidence comes from investigations of N-acetylgalactosaminyltransferase 5, encoded by GALNT5, a key enzyme that initiates mucin-type O-glycosylation and has been identified as a novel suppressor of ferroptosis in pancreatic adenocarcinoma [47]. GALNT5 knockdown significantly curtails the proliferation, migration, and invasion of pancreatic adenocarcinoma cells, while concurrently promoting ferroptosis, establishing a direct functional connection between the O-glycosylation machinery and ferroptotic cell death resistance [47].

The therapeutic implications of targeting mucin-type O-glycosylation for ferroptosis modulation are significant, particularly given the association between GALNT family enzymes, ferroptosis resistance, and immune cell infiltration [47]. The relationship between O-glycosylation and ferroptosis extends to tumor microenvironment interactions, where altered glycosylation patterns on cancer cell surfaces can influence the delivery of ferroptosis-inducing signals or protective factors [76]. Recent advances in O-glycoproteomics have enabled the identification of distinct glycosylation signatures associated with ferroptosis resistance across various cancer types, suggesting the potential for the development of O-glycosylation-based biomarkers and therapeutic strategies targeting this pathway [35].

### 4.3. GALNT14 and EGFR Pathway Regulation

O-linked glycosylation, catalyzed by the family of polypeptide GALNTs, is another important mechanism of ferroptosis regulation in cancer cells [48]. GALNT14 has emerged as a key regulator of both ferroptosis and apoptosis in ovarian cancer through its effects on epidermal growth factor receptor (EGFR) glycosylation and downstream mTOR pathway activation [48,77]. This enzyme regulates ferroptosis sensitivity through direct O-glycosylation of the EGFR, which stabilizes the protein and maintains its signaling activity, ultimately promoting cell survival pathways that inhibit ferroptotic cell death [48,49].

This mechanism involves GALNT14-mediated O-glycosylation of the EGFR, which affects receptor stability and signaling activity [48]. Downregulation of GALNT14 leads to reduced EGFR stability through modifications in O-glycosylation patterns, subsequently suppressing mTOR pathway activity and reducing the levels of key anti-ferroptotic proteins, including xCT and GPX4 [48,78]. This cascade ultimately results in enhanced sensitivity to both apoptosis and ferroptosis, suggesting that O-glycosylation modifications can coordinately regulate multiple cell death pathways [79].

The clinical relevance of GALNT14 in chemotherapy resistance has been demonstrated in cisplatin-resistant ovarian cancer models, in which combination therapy targeting both GALNT14 and mTOR signaling promoted cell death [48,80]. These findings suggest that O-glycosylation enzymes are viable therapeutic targets for overcoming ferroptosis resistance and that GALNT14 expression levels may serve as biomarkers for predicting treatment responses in ovarian cancer patients [77,81].

### 4.4. Other GALNT Family Enzymes in Cancer

The human polypeptide GALNT family comprises about 20 members, each with distinct substrate specificities and tissue expression patterns [35]. Several GALNT enzymes other than GALNT14 have been implicated in cancer progression and therapy resistance, although their specific roles in ferroptosis regulation remain unclear.

GALNT6 promotes oncogenic transformation and cancer cell growth through O-glycosylation of target proteins in colon and breast cancer cells, respectively [82,83]. Similarly, GALNT2 modifies EGFR activity in oral squamous cell carcinoma cells [50].

## 5. GAGs and Ferroptosis Protection

Previous studies have revealed the regulatory role of lipids in GPX4-mediated ferroptotic cancer cell death [7,84]. Recent studies have revealed a sophisticated mechanism by which cancer cells exploit sulfated GAGs to facilitate the cellular uptake of lipoproteins carrying potent antioxidants, thereby conferring ferroptosis resistance [51]. Functional genetic screens have identified GAG-dependent lipoprotein uptake as a critical determinant of ferroptosis sensitivity across diverse cancer types, including melanoma, pancreatic adenocarcinoma, HeLa, and clear cell renal cell carcinoma, with both low-density lipoprotein (LDL) and high-density lipoprotein (HDL), providing robust protection against ferroptotic cell death [51,84].

The protective effect primarily derives from lipoprotein delivery of α-tocopherol, delivered by both LDL and HDL and the most biologically active and abundant form of vitamin E, which functions as a potent lipophilic antioxidant that quenches lipid peroxyl radicals in cellular membranes [1,51]. The mechanism involves α-tocopherol metabolism to α-tocopherol hydroquinone, a highly active metabolite that inhibits 15-lipoxygenase and prevents lipid peroxidation cascades, driving ferroptotic cell death [1,85].

The cellular machinery centers on sulfated GAGs, including heparan sulfate and chondroitin sulfate, attached to cell-surface proteoglycans, such as syndecans and glypicans [86]. These sulfated GAG chains facilitate circulating lipoprotein uptake, with UDP-glucose 6-dehydrogenase (UGDH) functioning as the rate-limiting enzyme in GAG biosynthesis [51,87,88]. Enzymatic degradation studies using heparinases and chondroitinases have demonstrated that both heparan sulfate and chondroitin sulfate complement lipoprotein uptake and ferroptosis protection [51].

Clinical analysis of human clear cell renal cell carcinoma specimens revealed elevated chondroitin sulfate levels and increased lipoprotein-derived α-tocopherol compared with normal kidney tissue, suggesting the importance of this pathway in lipid-rich malignancies [51]. This GAG–lipoprotein axis represents a previously unrecognized extracellular mechanism for acquiring ferroptosis protection, distinct from intracellular systems such as GPX4 or FSP1, and may provide therapeutic opportunities for targeting ferroptosis-resistant cancers.

Despite Calhoon’s study, to our knowledge, no other studies have directly investigated the relationship between GAGs and ferroptosis, highlighting that this is an area that remains to be elucidated.

## 6. Future Directions

### 6.1. Targeting Glycosyltransferases in Cancer Therapy

The intersection of glycosylation and ferroptosis presents compelling opportunities for cancer therapy using multiple complementary approaches [60,89]. Directly targeting glycosyltransferases such as B3GNT3 in pancreatic cancer or ALG3 to induce immunogenic ferroptosis represents a promising strategy, particularly given the ability of ALG3 inhibition to enhance immunotherapy responses [41,42,59]. Although broad-spectrum glycosylation inhibitors such as tunicamycin provide proof of concept, the development of selective inhibitors with reduced toxicity to normal cells remains crucial for clinical translation [90,91].

The glycosylation–ferroptosis axis offers significant potential for combination therapies that exploit synthetic lethal interactions, including pairing glycosylation inhibitors with direct ferroptosis inducers such as GPX4 inhibitors or system Xc^−^ blockers [1,7,8]. Traditional chemotherapy and radiation therapy may also benefit from a combination of glycosylation-targeting approaches, as demonstrated by the inhibition of GALNT6 [49] and GALNT14 [48] expression in ovarian cancer. The immunogenic nature of ALG3-deficiency-induced ferroptosis particularly supports its combination with immune checkpoint inhibitors [42].

Targeting glycosylation to induce ferroptosis in cancer cells could potentially be achieved through cancer cell-targeted delivery of agents that modulate glycosylation pathways. The development of cancer cell-specific drug delivery strategies represents a critical advance in achieving precision therapeutics [92]. Engineering nanoparticles with tumor-targeting capabilities enables selective accumulation in cancer tissues while minimizing off-target effects [93]. Advanced delivery platforms that incorporate active targeting ligands, stimuli-responsive release mechanisms, and biomimetic designs can enhance the therapeutic index of glycosylation-targeting agents by ensuring preferential delivery to tumor cells [94,95].

The discovery of GAG-mediated lipoprotein uptake as a mechanism of ferroptosis resistance has opened up novel therapeutic avenues for targeting this previously unappreciated pathway [51]. Strategies include the enzymatic degradation of cell surface GAGs using heparinases and chondroitinases or small-molecule inhibitors of GAG biosynthesis enzymes, such as UGDH. Although enzymatic approaches show promise in preclinical studies, targeted delivery strategies are essential to minimize their effects on normal tissue GAGs.

### 6.2. Biomarker Development and Patient Stratification

The glycosylation status of ferroptosis regulators holds promise as a biomarker for predicting treatment responses and enabling precise oncology approaches [17,20]. The expression levels of key glycosyltransferases, such as B3GNT3, ALG3, and GALNT14, along with tumor GAG content and proteoglycan expression patterns, can guide patient stratification and therapeutic decision-making [41,42,96,97]. Advances in glycomics technologies and the development of circulating biomarkers offer additional opportunities for less invasive or non-invasive monitoring of treatment responses [96,97].

The clinical translation of glycosylation–ferroptosis therapeutics faces significant challenges due to the complexity and essential physiological roles of glycosylation pathways [33]. Key challenges include developing selective inhibitors with acceptable toxicity profiles, standardizing companion diagnostics for glycosylation-based biomarkers, and addressing the manufacturing complexities for enzymatic therapeutics [98,99,100]. The heterogeneity of glycosylation patterns within and between tumors further complicates biomarker development and clinical implementation.

Beyond the established targets, emerging therapeutic opportunities include glycosidases that remove protective sugar modifications, nucleotide sugar biosynthesis pathway enzymes, and chaperone systems involved in glycoprotein quality control [101]. The intersection of glycosylation with other post-translational modifications, such as O-linked glycosylation, offers additional opportunities for precision targeting [44,74]. Collectively, these diverse approaches represent a new therapeutic paradigm that can transform cancer treatment by exploiting the fundamental dependence of cancer cell survival on glycosylation-mediated ferroptosis resistance mechanisms.

### 6.3. Research Opportunities

The development of new technologies for studying glycosylation–ferroptosis interactions will be crucial in advancing the field. A combined approach of genomics and glycomics may enable revealing the regulation of glycosylation [96]. Chemical biology approaches for selectively modifying specific glycan structures or glycoproteins could provide more precise tools for dissecting mechanistic relationships. Imaging technologies for visualizing glycosylation in real time could provide valuable insights into the dynamics of these processes [97]. Fluorescent probes for specific glycan structures or ferroptosis markers, combined with advanced microscopy techniques, could enable detailed characterization of spatial and temporal relationships.

The complexity of glycosylation–ferroptosis interactions necessitates systems-level approaches for comprehensive understanding [63,102]. Integrated omics approaches combining glycomics, proteomics, metabolomics, and transcriptomics could provide insights into the global effects of glycosylation on ferroptosis regulation. Computational modeling of glycosylation networks and their effects on ferroptosis could help identify key regulatory nodes and predict the effects of therapeutic interventions [63,103]. Systems biology approaches could be particularly valuable in identifying patterns in complex glycosylation.

## 7. Conclusions

The oxidative stress landscape in cancer cells provides an additional layer of complexity in glycosylation–ferroptosis interactions. The elevated ROS levels characteristic of cancer cells, combined with their metabolic reprogramming, create a cellular environment where glycosylation-mediated protection against ferroptosis becomes even more critical for survival. Understanding how glycosylation modifications intersect with oxidative stress pathways will be essential in developing targeted therapeutic strategies that can effectively exploit the redox vulnerabilities of cancer cells while minimizing toxicity for normal tissues.

The intersection of glycosylation and ferroptosis represents a rapidly emerging area in cancer biology with significant therapeutic implications. The evidence reviewed here demonstrates that specific glycosylation modifications can profoundly influence cellular susceptibility to ferroptotic cell death through diverse mechanisms, ranging from direct effects on ferroptosis regulators to indirect modulation via metabolic pathways and antioxidant uptake. N-glycosylation of 4F2hc by B3GNT3, immunogenic ferroptosis induced by ALG3 deficiency, and the GAG-mediated lipoprotein uptake pathway exemplify the sophisticated ways in which cancer cells exploit the glycosylation machinery to evade ferroptotic cell death.

These findings have important implications for cancer therapy, as they identify new targets for inducing ferroptosis and overcoming treatment resistance. The ability to modulate ferroptosis sensitivity through glycosylation provides opportunities for developing combination therapies that exploit synthetic lethal interactions. Furthermore, the immunogenic nature of glycosylation deficiency-induced ferroptosis highlights promising strategies for enhancing immunotherapy responses.

However, several key challenges remain in translating these findings into clinical applications. The development of selective glycosyltransferase inhibitors, cancer cell-targeted delivery of glycosylation-modulatory drugs, the identification of predictive biomarkers, the optimization of combination therapeutic approaches, and combinatorial research approaches are crucial in clinical success. Additionally, a deeper understanding of the mechanistic basis of glycosylation–ferroptosis interactions is necessary to guide rational therapeutic design.

As our understanding of the glycosylation–ferroptosis axis continues to evolve, it is becoming clear that this intersection represents more than just an interesting biological observation; it is a fundamental aspect of cancer cell biology with the potential to transform therapeutic approaches. The ability to manipulate cellular susceptibility to ferroptosis through targeted modulation of specific glycosylation pathways provides a new paradigm for cancer treatment that complements existing therapeutic modalities while opening up entirely new avenues for intervention.

The therapeutic potential of targeting the glycosylation–ferroptosis axis extends beyond the direct cytotoxic effects. The modulation of tumor immunogenicity through glycosylation changes could enhance the efficacy of immunotherapies, whereas the disruption of metabolic adaptations could overcome therapy resistance. As we continue to unravel the complexities of this intersection, new therapeutic opportunities will emerge, offering hope for improved outcomes in cancer patients.

## Figures and Tables

**Table 1:** Summary of ferroptosis regulation-associated genes and/or proteins. Abbreviations: EGFR, epidermal growth factor receptor; HCC, hepatocellular carcinoma; GAG, glycosaminoglycan; GlcNAc, N-acetylglucosamine; SREBP, sterol-regulated element-binding protein; PDAC, pancreatic ductal carcinoma; TFRC, transferrin receptor.

Gene or Protein	Function Related to Ferroptosis	Cancer Type	Reference
B3GNT3	Stabilizes 4F2hc through N-glycosylation, enhances system Xc^−^ activity, confers ferroptosis resistance	PDAC, in vitro	[41]
4F2hc (SLC3A2)	Heavy chain subunit of system Xc^−^, essential in cystine uptake and glutathione biosynthesis	PDAC, in vitro	[41]
ALG3	Catalyzes mannosylation in N-glycan assembly; inhibition leads to lipid accumulation through SREBP1-dependent lipogenesis	Multiple cancer types (renal cortical adenocarcinoma, colorectal adenocarcinoma, melanoma, breast cancer), in vivo	[42]
EXT2	Forms complex with EXT1 for heparan sulfate synthesis; depletion reduces transsulfuration pathway activity, enhances ferroptosis sensitivity	Glioblastoma, in vitro	[43]
EXT1	Partner of EXT2 in heparan sulfate chain polymerization	Glioblastoma, in vitro	[43]
TFRC	O-GlcNAcylation at Ser687 regulates ubiquitination and stability; controls iron accumulation and ferroptosis sensitivity	HCC, in vitro	[44]
MARCH8	Ubiquitin E3 ligase that targets TFRC; binding decreased by de-O-GlcNAcylation	HCC, in vitro	[44]
HUWE1	HECT domain ubiquitin ligase that degrades TFRC, protects from ferroptosis	Hepatocytes (acute liver injury), in vitro	[45]
YAP	O-GlcNAcylation enhances transcription of TFRC, increases ferroptosis sensitivity	HCC, in vitro	[46]
GALNT5	Initiates mucin-type O-glycosylation; knockdown promotes ferroptosis	Pancreatic adenocarcinoma, in vivo	[47]
GALNT14	mTOR signaling; downregulation enhances ferroptosis and apoptosis sensitivity	Ovarian cancer, in vivo	[48]
GALNT6	Promotes oncogenic transformation through O-glycosylation	Colon and breast cancer, in vitro	[49]
GALNT2	Modifies EGFR activity	Oral squamous cell carcinoma, in vitro	[50]
UGDH	Rate-limiting enzyme in GAG biosynthesis; facilitates lipoprotein uptake and α-tocopherol delivery for ferroptosis protection	Multiple cancer types (melanoma, pancreatic adenocarcinoma, HeLa, clear cell renal cell carcinoma), in vivo	[51]

## Data Availability

Not applicable.

## Abbreviations

The following abbreviations are used in this manuscript:

ACSL	acyl-CoA synthetase long-chain family member
ALG	alpha-1,3-mannosyltransferase
CD	cluster of differentiation
EGFR	epidermal growth factor receptor
ER	endoplasmic reticulum
EXT	exostosin glycosyltransferase
EXTL	exostosin-like glycosyltransferase
FSP	ferroptosis suppressor protein
GAG	glycosaminoglycan
GALNT	N-acetylgalactosaminyltransferase
GPX	glutathione peroxidase
HCC	hepatocellular carcinoma
HUWE	HECT domain-containing ubiquitin ligase E3 ligase
GlcNAc	N-acetylglucosamine
MARCH	membrane-associated RING-CH
O-GlcNAcylation	O-linked β-N-acetylglucosaminylation
OGT	O-linked β-N-acetylglucosamine transferase
PDAC	pancreatic ductal adenocarcinoma
PUFA	polyunsaturated fatty acid
ROS	reactive oxygen species
SREBP	sterol-regulated element-binding protein
TFRC	transferrin receptor
UGDH	UDP-glucose 6-dehydrogenase

## References

[1] Stockwell B.R., Friedmann Angeli J.P., Bayir H., Bush A.I., Conrad M., Dixon S.J., Fulda S., Gascón S., Hatzios S.K., Kagan V.E. (2017). Ferroptosis: A Regulated Cell Death Nexus Linking Metabolism, Redox Biology, and Disease. Cell.

[2] Dixon S.J., Lemberg K.M., Lamprecht M.R., Skouta R., Zaitsev E.M., Gleason C.E., Patel D.N., Bauer A.J., Cantley A.M., Yang W.S. (2012). Ferroptosis: An Iron-Dependent Form of Nonapoptotic Cell Death. Cell.

[3] Jiang X., Stockwell B.R., Conrad M. (2021). Ferroptosis: Mechanisms, Biology and Role in Disease. Nat. Rev. Mol. Cell Biol..

[4] Sies H., Berndt C., Jones D.P. (2017). Oxidative Stress. Annu. Rev. Biochem..

[5] Schieber M., Chandel N.S. (2014). ROS Function in Redox Signaling and Oxidative Stress. Curr. Biol..

[6] Zheng J., Conrad M. (2020). The Metabolic Underpinnings of Ferroptosis. Cell Metab..

[7] Yang W.S., Sriramaratnam R., Welsch M.E., Shimada K., Skouta R., Viswanathan V.S., Cheah J.H., Clemons P.A., Shamji A.F., Clish C.B. (2014). Regulation of Ferroptotic Cancer Cell Death by GPX4. Cell.

[8] Koppula P., Zhuang L., Gan B. (2021). Cystine Transporter SLC7A11/XCT in Cancer: Ferroptosis, Nutrient Dependency, and Cancer Therapy. Protein Cell.

[9] Bersuker K., Hendricks J.M., Li Z., Magtanong L., Ford B., Tang P.H., Roberts M.A., Tong B., Maimone T.J., Zoncu R. (2019). The CoQ Oxidoreductase FSP1 Acts Parallel to GPX4 to Inhibit Ferroptosis. Nature.

[10] Doll S., Freitas F.P., Shah R., Aldrovandi M., da Silva M.C., Ingold I., Grocin A.G., Xavier da Silva T.N., Panzilius E., Scheel C.H. (2019). FSP1 Is a Glutathione-Independent Ferroptosis Suppressor. Nature.

[11] Nakamura T., Hipp C., Santos Dias Mourão A., Borggräfe J., Aldrovandi M., Henkelmann B., Wanninger J., Mishima E., Lytton E., Emler D. (2023). Phase Separation of FSP1 Promotes Ferroptosis. Nature.

[12] Mao C., Liu X., Zhang Y., Lei G., Yan Y., Lee H., Koppula P., Wu S., Zhuang L., Fang B. (2021). DHODH-Mediated Ferroptosis Defence Is a Targetable Vulnerability in Cancer. Nature.

[13] Li Y., Ran Q., Duan Q., Jin J., Wang Y., Yu L., Wang C., Zhu Z., Chen X., Weng L. (2024). 7-Dehydrocholesterol Dictates Ferroptosis Sensitivity. Nature.

[14] Freitas F.P., Alborzinia H., dos Santos A.F., Nepachalovich P., Pedrera L., Zilka O., Inague A., Klein C., Aroua N., Kaushal K. (2024). 7-Dehydrocholesterol Is an Endogenous Suppressor of Ferroptosis. Nature.

[15] Liang D., Feng Y., Zandkarimi F., Wang H., Zhang Z., Kim J., Cai Y., Gu W., Stockwell B.R., Jiang X. (2023). Ferroptosis Surveillance Independent of GPX4 and Differentially Regulated by Sex Hormones. Cell.

[16] Munkley J., Elliott D.J., Munkley J., Elliott D.J. (2016). Hallmarks of Glycosylation in Cancer. Oncotarget.

[17] Pinho S.S., Reis C.A. (2015). Glycosylation in Cancer: Mechanisms and Clinical Implications. Nat. Rev. Cancer.

[18] Taniguchi N., Kizuka Y. (2015). Glycans and Cancer: Role of N-Glycans in Cancer Biomarker, Progression and Metastasis, and Therapeutics. Adv. Cancer Res..

[19] Gama C.I., Tully S.E., Sotogaku N., Clark P.M., Rawat M., Vaidehi N., Goddard W.A., Nishi A., Hsieh-Wilson L.C. (2006). Sulfation Patterns of Glycosaminoglycans Encode Molecular Recognition and Activity. Nat. Chem. Biol..

[20] Reily C., Stewart T.J., Renfrow M.B., Novak J. (2019). Glycosylation in Health and Disease. Nat. Rev. Nephrol..

[21] Peixoto A., Relvas-Santos M., Azevedo R., Lara Santos L., Ferreira J.A. (2019). Protein Glycosylation and Tumor Microenvironment Alterations Driving Cancer Hallmarks. Front. Oncol..

[22] Stanczak M.A., Mantuano N.R., Kirchhammer N., Sanin D.E., Jacob F., Coelho R., Everest-Dass A.V., Wang J., Trefny M.P., Monaco G. (2022). Targeting Cancer Glycosylation Repolarizes Tumor-Associated Macrophages Allowing Effective Immune Checkpoint Blockade. Sci. Transl. Med..

[23] Pu Y., Zhou B., Bing J., Wang L., Chen M., Shen Y., Gao S., Zhou M., Wu W., Shi J. (2024). Ultrasound-Triggered and Glycosylation Inhibition-Enhanced Tumor Piezocatalytic Immunotherapy. Nat. Commun..

[24] Sørensen D.M., Büll C., Madsen T.D., Lira-Navarrete E., Clausen T.M., Clark A.E., Garretson A.F., Karlsson R., Pijnenborg J.F.A., Yin X. (2023). Identification of Global Inhibitors of Cellular Glycosylation. Nat. Commun..

[25] Zhang H., Ma J., Hou C., Luo X., Zhu S., Peng Y., Peng C., Li P., Meng H., Xia Y. (2025). A ROS-Mediated Oxidation-O-GlcNAcylation Cascade Governs Ferroptosis. Nat. Cell Biol..

[26] Zhao J., Lang M. (2023). New Insight into Protein Glycosylation in the Development of Alzheimer’s Disease. Cell Death Discov..

[27] Trachootham D., Alexandre J., Huang P. (2009). Targeting Cancer Cells by ROS-Mediated Mechanisms: A Radical Therapeutic Approach?. Nat. Rev. Drug Discov..

[28] Doll S., Proneth B., Tyurina Y.Y., Panzilius E., Kobayashi S., Ingold I., Irmler M., Beckers J., Aichler M., Walch A. (2017). ACSL4 Dictates Ferroptosis Sensitivity by Shaping Cellular Lipid Composition. Nat. Chem. Biol..

[29] Yan H., Zou T., Tuo Q., Xu S., Li H., Belaidi A.A., Lei P. (2021). Ferroptosis: Mechanisms and Links with Diseases. Signal Transduct. Target. Ther..

[30] Wang W., Green M., Choi J.E., Gijón M., Kennedy P.D., Johnson J.K., Liao P., Lang X., Kryczek I., Sell A. (2019). CD8+ T Cells Regulate Tumour Ferroptosis during Cancer Immunotherapy. Nature.

[31] He M., Zhou X., Wang X. (2024). Glycosylation: Mechanisms, Biological Functions and Clinical Implications. Signal Transduct. Target. Ther..

[32] Hart G.W., Copeland R.J. (2010). Glycomics Hits the Big Time. Cell.

[33] Ohtsubo K., Marth J.D. (2006). Glycosylation in Cellular Mechanisms of Health and Disease. Cell.

[34] Khoury G.A., Baliban R.C., Floudas C.A. (2011). Proteome-Wide Post-Translational Modification Statistics: Frequency Analysis and Curation of the Swiss-Prot Database. Sci. Rep..

[35] Schjoldager K.T., Narimatsu Y., Joshi H.J., Clausen H. (2020). Global View of Human Protein Glycosylation Pathways and Functions. Nat. Rev. Mol. Cell Biol..

[36] Kellokumpu S., Hassinen A., Glumoff T. (2015). Glycosyltransferase Complexes in Eukaryotes: Long-Known, Prevalent but Still Unrecognized. Cell. Mol. Life Sci..

[37] Ramírez A.S., Locher K.P. (2023). Structural and Mechanistic Studies of the N-Glycosylation Machinery: From Lipid-Linked Oligosaccharide Biosynthesis to Glycan Transfer. Glycobiology.

[38] Varki A., Cummings R.D., Esko J.D., Stanley P., Hart G.W., Aebi M., Mohnen D., Kinoshita T., Packer N.H., Prestegard J.H. (2022). Essentials of Glycobiology.

[39] Hetz C., Zhang K., Kaufman R.J. (2020). Mechanisms, Regulation and Functions of the Unfolded Protein Response. Nat. Rev. Mol. Cell Biol..

[40] Tabas I., Ron D. (2011). Integrating the Mechanisms of Apoptosis Induced by Endoplasmic Reticulum Stress. Nat. Cell Biol..

[41] Ma H., Chen X., Mo S., Zhang Y., Mao X., Chen J., Liu Y., Tong W.M., Lu Z., Yu S. (2023). Targeting N-Glycosylation of 4F2hc Mediated by Glycosyltransferase B3GNT3 Sensitizes Ferroptosis of Pancreatic Ductal Adenocarcinoma. Cell Death Differ..

[42] Liu P., Lin C., Liu Z., Zhu C., Lin Z., Xu D., Chen J., Huang Q., Li C.Y., Hou L. (2022). Inhibition of ALG3 Stimulates Cancer Cell Immunogenic Ferroptosis to Potentiate Immunotherapy. Cell. Mol. Life Sci..

[43] Matesanz-Sánchez R., Peitzsch M., Lange I., Mircetic J., Seifert M., Cordes N., Vehlow A. (2025). A Novel Role of Exostosin Glycosyltransferase 2 (EXT2) in Glioblastoma Cell Metabolism, Radiosensitivity and Ferroptosis. Cell Death Differ..

[44] Zhou X., Wang Y., Li X., Zhou J., Yang W., Wang X., Jiao S., Zuo W., You Z., Ying W. (2024). O-GlcNAcylation Regulates the Stability of Transferrin Receptor (TFRC) to Control the Ferroptosis in Hepatocellular Carcinoma Cells. Redox Biol..

[45] Wu Y., Jiao H., Yue Y., He K., Jin Y., Zhang J., Zhang J., Wei Y., Luo H., Hao Z. (2022). Ubiquitin Ligase E3 HUWE1/MULE Targets Transferrin Receptor for Degradation and Suppresses Ferroptosis in Acute Liver Injury. Cell Death Differ..

[46] Zhu G., Murshed A., Li H., Ma J., Zhen N., Ding M., Zhu J., Mao S., Tang X., Liu L. (2021). O-GlcNAcylation Enhances Sensitivity to RSL3-Induced Ferroptosis via the YAP/TFRC Pathway in Liver Cancer. Cell Death Discov..

[47] Yan J., Gong H., Han S., Liu J., Wu Z., Wang Z., Wang T. (2023). GALNT5 Functions as a Suppressor of Ferroptosis and a Predictor of Poor Prognosis in Pancreatic Adenocarcinoma. Am. J. Cancer Res..

[48] Li H.W., Liu M.B., Jiang X., Song T., Feng S.X., Wu J.Y., Deng P.F., Wang X.Y. (2022). GALNT14 Regulates Ferroptosis and Apoptosis of Ovarian Cancer through the EGFR/MTOR Pathway. Future Oncol..

[49] Lin T.-C., Chen S.-T., Huang M.-C., Huang J., Hsu C.-L., Juan H.-F., Lin H.-H., Chen C.-H., Lin T.-C., Chen S.-T. (2017). GALNT6 Expression Enhances Aggressive Phenotypes of Ovarian Cancer Cells by Regulating EGFR Activity. Oncotarget.

[50] Lin M.C., Huang M.J., Liu C.H., Yang T.L., Huang M.C. (2014). GALNT2 Enhances Migration and Invasion of Oral Squamous Cell Carcinoma by Regulating EGFR Glycosylation and Activity. Oral. Oncol..

[51] Calhoon D., Sang L., Ji F., Bezwada D., Hsu S.C., Cai F., Kim N., Basu A., Wu R., Pimentel A. (2025). Glycosaminoglycan-Driven Lipoprotein Uptake Protects Tumours from Ferroptosis. Nature.

[52] Daher B., Vučetić M., Pouysségur J. (2020). Cysteine Depletion, a Key Action to Challenge Cancer Cells to Ferroptotic Cell Death. Front. Oncol..

[53] Yan R., Xie E., Li Y., Li J., Zhang Y., Chi X., Hu X., Xu L., Hou T., Stockwell B.R. (2022). The Structure of Erastin-Bound XCT–4F2hc Complex Reveals Molecular Mechanisms Underlying Erastin-Induced Ferroptosis. Cell Res..

[54] Rapozzi V., Comuzzi C., Di Giorgio E., Xodo L.E. (2025). KRAS and NRF2 Drive Metabolic Reprogramming in Pancreatic Cancer Cells: The Influence of Oxidative and Nitrosatice Stress. Front. Cell Dev. Biol..

[55] Kong K., Zhao Y., Xia L., Jiang H., Xu M., Zheng J. (2020). B3GNT3: A Prognostic Biomarker Associated with Immune Cell Infiltration in Pancreatic Adenocarcinoma. Oncol. Lett..

[56] Schwarz F., Aebi M. (2011). Mechanisms and Principles of N-Linked Protein Glycosylation. Curr. Opin. Struct. Biol..

[57] Freeze H.H. (2006). Genetic Defects in the Human Glycome. Nat. Rev. Genet..

[58] Conrad M., Pratt D.A. (2019). The Chemical Basis of Ferroptosis. Nat. Chem. Biol..

[59] Hassannia B., Vandenabeele P., Vanden Berghe T. (2019). Targeting Ferroptosis to Iron Out Cancer. Cancer Cell.

[60] Chen X., Kang R., Kroemer G., Tang D. (2021). Broadening Horizons: The Role of Ferroptosis in Cancer. Nat. Rev. Clin. Oncol..

[61] Galluzzi L., Vitale I., Warren S., Adjemian S., Agostinis P., Martinez A.B., Chan T.A., Coukos G., Demaria S., Deutsch E. (2020). Consensus Guidelines for the Definition, Detection and Interpretation of Immunogenic Cell Death. J. Immunother. Cancer.

[62] Tang D., Chen X., Kang R., Kroemer G. (2021). Ferroptosis: Molecular Mechanisms and Health Implications. Cell Res..

[63] Neelamegham S., Mahal L.K. (2016). Multi-Level Regulation of Cellular Glycosylation: From Genes to Transcript to Enzyme to Structure. Curr. Opin. Struct. Biol..

[64] Busse M., Feta A., Presto J., Wilén M., Grønning M., Kjellén L., Kusche-Gullberg M. (2007). Contribution of EXT1, EXT2, and EXTL3 to Heparan Sulfate Chain Elongation. J. Biol. Chem..

[65] Leisico F., Omeiri J., Le Narvor C., Beaudouin J., Hons M., Fenel D., Schoehn G., Couté Y., Bonnaffé D., Sadir R. (2022). Structure of the Human Heparan Sulfate Polymerase Complex EXT1-EXT2. Nat. Commun..

[66] Li H., Chapla D., Amos R.A., Ramiah A., Moremen K.W., Li H. (2023). Structural Basis for Heparan Sulfate Co-Polymerase Action by the EXT1–2 Complex. Nat. Chem. Biol..

[67] Nadanaka S., Zhou S., Kagiyama S., Shoji N., Sugahara K., Sugihara K., Asano M., Kitagawa H. (2013). EXTL2, a Member of the EXT Family of Tumor Suppressors, Controls Glycosaminoglycan Biosynthesis in a Xylose Kinase-Dependent Manner. J. Biol. Chem..

[68] Hayano M., Yang W.S., Corn C.K., Pagano N.C., Stockwell B.R. (2016). Loss of Cysteinyl-TRNA Synthetase (CARS) Induces the Transsulfuration Pathway and Inhibits Ferroptosis Induced by Cystine Deprivation. Cell Death Differ..

[69] Zhang H.F., Klein Geltink R.I., Parker S.J., Sorensen P.H. (2022). Transsulfuration, Minor Player or Crucial for Cysteine Homeostasis in Cancer. Trends Cell Biol..

[70] Sehm T., Rauh M., Wiendieck K., Buchfelder M., Eyüpoglu I.Y., Savaskan N.E. (2016). Temozolomide Toxicity Operates in a XCT/SLC7a11 Dependent Manner and Is Fostered by Ferroptosis. Oncotarget.

[71] Upadhyayula P.S., Higgins D.M., Mela A., Banu M., Dovas A., Zandkarimi F., Patel P., Mahajan A., Humala N., Nguyen T.T.T. (2023). Dietary Restriction of Cysteine and Methionine Sensitizes Gliomas to Ferroptosis and Induces Alterations in Energetic Metabolism. Nat. Commun..

[72] Lee Y.S., Lee D.H., Choudry H.A., Bartlett D.L., Lee Y.J. (2018). Ferroptosis-Induced Endoplasmic Reticulum Stress: Crosstalk Between Ferroptosis and Apoptosis. Mol. Cancer Res..

[73] Thompson N., Wakarchuk W. (2022). O-Glycosylation and Its Role in Therapeutic Proteins. Biosci. Rep..

[74] Yang X., Qian K. (2017). Protein O-GlcNAcylation: Emerging Mechanisms and Functions. Nat. Rev. Mol. Cell Biol..

[75] Oliveira-Ferrer L., Legler K., Milde-Langosch K. (2017). Role of Protein Glycosylation in Cancer Metastasis. Semin. Cancer Biol..

[76] Rodrigues J.G., Balmaña M., Macedo J.A., Poças J., Fernandes Â., de-Freitas-Junior J.C.M., Pinho S.S., Gomes J., Magalhães A., Gomes C. (2018). Glycosylation in Cancer: Selected Roles in Tumour Progression, Immune Modulation and Metastasis. Cell Immunol..

[77] Chen B., Zhao L., Yang R., Xu T. (2023). The Recent Advancements of Ferroptosis in the Diagnosis, Treatment and Prognosis of Ovarian Cancer. Front. Genet..

[78] Zhang Y., Swanda R.V., Nie L., Liu X., Wang C., Lee H., Lei G., Mao C., Koppula P., Cheng W. (2021). MTORC1 Couples Cyst(e)Ine Availability with GPX4 Protein Synthesis and Ferroptosis Regulation. Nat. Commun..

[79] Liu Y., Wang Y., Liu J., Kang R., Tang D. (2021). Interplay between MTOR and GPX4 Signaling Modulates Autophagy-Dependent Ferroptotic Cancer Cell Death. Cancer Gene Ther..

[80] Kapper C., Oppelt P., Arbeithuber B., Gyunesh A.A., Vilusic I., Stelzl P., Rezk-Füreder M. (2024). Targeting Ferroptosis in Ovarian Cancer: Novel Strategies to Overcome Chemotherapy Resistance. Life Sci..

[81] Liao H., Wang Y., Zou L., Fan Y., Wang X., Tu X., Zhu Q., Wang J., Liu X., Dong C. (2024). Relationship of MTORC1 and Ferroptosis in Tumors. Discov. Oncol..

[82] Park J.H., Nishidate T., Kijima K., Ohashi T., Takegawa K., Fujikane T., Hirata K., Nakamura Y., Katagiri T. (2010). Critical Roles of Mucin 1 Glycosylation by Transactivated Polypeptide N-Acetylgalactosaminyltransferase 6 in Mammary Carcinogenesis. Cancer Res..

[83] Lavrsen K., Dabelsteen S., Vakhrushev S.Y., Levann A.M.R., Haue A.D., Dylander A., Mandel U., Hansen L., Frodin M., Bennett E.P. (2018). De Novo Expression of Human Polypeptide N-Acetylgalactosaminyltransferase 6 (GalNAc-T6) in Colon Adenocarcinoma Inhibits the Differentiation of Colonic Epithelium. J. Biol. Chem..

[84] Garcia-Bermudez J., Baudrier L., Bayraktar E.C., Shen Y., La K., Guarecuco R., Yucel B., Fiore D., Tavora B., Freinkman E. (2019). Squalene Accumulation in Cholesterol Auxotrophic Lymphomas Prevents Oxidative Cell Death. Nature.

[85] Shah R., Shchepinov M.S., Pratt D.A. (2018). Resolving the Role of Lipoxygenases in the Initiation and Execution of Ferroptosis. ACS Cent. Sci..

[86] Iozzo R.V., Schaefer L. (2015). Proteoglycan Form and Function: A Comprehensive Nomenclature of Proteoglycans. Matrix Biol..

[87] Zimmer B.M., Barycki J.J., Simpson M.A. (2021). Integration of Sugar Metabolism and Proteoglycan Synthesis by UDP-Glucose Dehydrogenase. J. Histochem. Cytochem..

[88] Price M.J., Nguyen A.D., Byemerwa J.K., Flowers J., Baëta C.D., Goodwin C.R., Price M.J., Nguyen A.D., Byemerwa J.K., Flowers J. (2023). UDP-Glucose Dehydrogenase (UGDH) in Clinical Oncology and Cancer Biology. Oncotarget.

[89] Büll C., Boltje T.J., Balneger N., Weischer S.M., Wassink M., Van Gemst J.J., Bloemendal V.R., Boon L., Van Der Vlag J., Heise T. (2018). Sialic Acid Blockade Suppresses Tumor Growth by Enhancing T-Cell-Mediated Tumor Immunity. Cancer Res..

[90] Rillahan C.D., Antonopoulos A., Lefort C.T., Sonon R., Azadi P., Ley K., Dell A., Haslam S.M., Paulson J.C. (2012). Global Metabolic Inhibitors of Sialyl- and Fucosyltransferases Remodel the Glycome. Nat. Chem. Biol..

[91] Hudak J.E., Bertozzi C.R. (2014). Glycotherapy: New Advances Inspire a Reemergence of Glycans in Medicine. Chem. Biol..

[92] Rosenblum D., Joshi N., Tao W., Karp J.M., Peer D. (2018). Progress and Challenges towards Targeted Delivery of Cancer Therapeutics. Nat. Commun..

[93] Mitchell M.J., Billingsley M.M., Haley R.M., Wechsler M.E., Peppas N.A., Langer R. (2020). Engineering Precision Nanoparticles for Drug Delivery. Nat. Rev. Drug Discov..

[94] Fan D., Cao Y., Cao M., Wang Y., Cao Y., Gong T. (2023). Nanomedicine in Cancer Therapy. Signal Transduct. Target. Ther..

[95] Rennick J.J., Johnston A.P.R., Parton R.G. (2021). Key Principles and Methods for Studying the Endocytosis of Biological and Nanoparticle Therapeutics. Nat. Nanotechnol..

[96] Lauc G., Essafi A., Huffman J.E., Hayward C., Knežević A., Kattla J.J., Polašek O., Gornik O., Vitart V., Abrahams J.L. (2010). Genomics Meets Glycomics—The First GWAS Study of Human N-Glycome Identifies HNF1α as a Master Regulator of Plasma Protein Fucosylation. PLoS Genet..

[97] Laughlin S.T., Bertozzi C.R. (2009). Imaging the Glycome. Proc. Natl. Acad. Sci. USA.

[98] Almahayni K., Spiekermann M., Fiore A., Yu G., Pedram K., Möckl L. (2022). Small Molecule Inhibitors of Mammalian Glycosylation. Matrix Biol. Plus.

[99] Ivanova A., Falcioni F. (2022). Challenges and Opportunities for the Large-Scale Chemoenzymatic Glycoengineering of Therapeutic N-Glycosylated Monoclonal Antibodies. Front. Catal..

[100] Kang J.G., Ko J.H., Kim Y.S. (2011). Pros and Cons of Using Aberrant Glycosylation as Companion Biomarkers for Therapeutics in Cancer. BMB Rep..

[101] Gloster T.M., Vocadlo D.J. (2012). Developing Inhibitors of Glycan Processing Enzymes as Tools for Enabling Glycobiology. Nat. Chem. Biol..

[102] Mardinoglu A., Nielsen J. (2015). New Paradigms for Metabolic Modeling of Human Cells. Curr. Opin. Biotechnol..

[103] Krambeck F.J., Bennun S.V., Narang S., Choi S., Yarema K.J., Betenbaugh M.J. (2009). A Mathematical Model to Derive N-Glycan Structures and Cellular Enzyme Activities from Mass Spectrometric Data. Glycobiology.

